# Editorial: Plant systems biology: integration of system-wide studies to elucidate central features in biological processes

**DOI:** 10.3389/fpls.2023.1324837

**Published:** 2023-11-07

**Authors:** Josefa M. Alamillo, Antoni Garcia-Molina

**Affiliations:** ^1^ Departamento de Botánica, Ecología y Fisiología Vegetal, Campus de Excelencia Internacional Agroalimentario, CEIA3, Universidad de Córdoba, Córdoba, Spain; ^2^ Centre for Research in Agricultural Genomics, Spanish National Research Council (CSIC), Barcelona, Spain

**Keywords:** systems biology, high throughput analysis, network analysis, integrative analysis, bioinformatics & computational biology

Plant homeostasis relies on sophisticated interplays and cross-talks among biological components that ultimately configure systemic networks that tailor growth and development programs and mediate acclimation to environmental fluctuations. The generalisation and diversification of high-throughput methodologies for transcriptome, proteome, and metabolome profiling along with the implementation of *ad hoc* tools for the analysis permitted to address system-wide investigations of molecular responses. In this context, Systems Biology emerged as the discipline that integrates large-scale data generation and analysis to provide holistic interpretations of biological questions. In the field of plant science, systemic studies focus on to gain insight into how plants integrate and regulate environmental cues, respond to stressors, or sustain developmental events, among all. More recently, the large volume of available datasets is allowing the implementation of artificial intelligence-based algorithms to train models that anticipate how plants would respond according to the existing knowledge.

Despite the expansion in the usage of system-wide technologies to investigate plant biological processes, the enormous potential to provide systemic interpretations is still being explored. In this regard, Systems Biology includes versatile strategies to integrate complex datasets ranging from differences in molecular abundances to co-expression and co-regulation networks or predictive models that contribute to generate knowledge from holistic perspectives and anticipate trajectories for molecular responses. The adoption of systemic analysis of high-throughput data and modelling paves the way for proper investigation of plant biological responses to frequent environmental cues or challenges, taking into consideration the whole organism.

The present Research Topic includes a series of exemplary studies that addressed systemic questions in the field of plant biology using multi-disciplinary and state-of-the-art methods for high-throughput profiling and analysis that ultimately provided a general interpretation of homeostatic responses.

Xu et al. conducted a comprehensive multi-level transcriptome-wide analysis of dynamic responses to drought in the model plant *Arabidopsis thaliana*. By means of RNA-sequencing on ribosomal-depleted RNA samples the authors addressed the transcriptional reconfigurations of both nuclear and organellar (chloroplast and mitochondrial) encoded transcripts and monitored post-transcriptional events, namely editing efficiency and alternative splicing (AS). Direct comparison of the differences in transcript abundance detected in their work with previous results from a referent study derived from microarrays permitted the identification of central features in the response to drought. In addition, the authors found out that about 50% of the AS events under drought treatments took place in transcripts that did not display changes in abundance. Follow-up on mutant lines with abrogated expression for candidate genes that only underwent alternative splicing under drought resulted in tolerance to water withdrawal *in planta*. Therefore, this work highlights the necessity to integrate different levels of transcriptome analysis since the responses described at post-transcriptional levels would have been overlooked in conventional approaches exclusively focused on differences in steady-state transcript levels.

Systems Biology also addresses the integration different large-scale datasets to elaborate models to interpret, and even predict, the mechanisms underpinning biological responses. In the study by Punyasu et al. the authors combined transcriptome and metabolome datasets to elaborate a constraint-based model to explain how *Manihot esculenta* (cassava) leaves address systemic metabolic responses to drought episodes. The model unveiled the relevance of the phospho*enol*pyruvate carboxylase (PEPC) during drought due to its pivotal role to locally concentrating CO_2_, facilitate its fixation by RuBisCO and ultimately increase sugar production to cope with water withdrawal. PEPC is accordingly proposed as a candidate to reinforce abiotic stress tolerance in other species of agronomical interest, although likely existing trade-off for plant biomass losses is discussed.

The potential of high-throughput technologies can be used to address biological questions in unexplored species of interest, where *bona fide* biological information is lacking. Zhou et al. employed high-resolution sequencing methods to reconstruct *de novo* the genome of the moss *Niphotrichum japonicum* and thus investigate the systemic mechanism conferring tolerance to heat stress. Synteny and comparative analysis pointed to the organisation of *N, japonicum* genome in 14 pseudochromosomes with a comparable structure to other mosses and, based on functional annotations, uncovered a specific coreset of genes likely related to adaptation to high temperatures. Further transcriptome analysis qualified one-third of the total number of differentially expressed genes showing that most of them have equivalent functions in heat stress responses in other plant species. Finally, Weighted Correlation Network Analysis (WGCNA) was employed as a systemic strategy to capture the holistic mechanisms behind the stress response and moreover extract the hubs that were proposed to act as central features mediating resilience to heat.

Another example of the potential WGCNA to retrieve potential candidates genes involved in biological processes was provided by the study of Zhao et al., where the authors interrogated which genes are key for the lignin production in *Populus trichocarpa.* Networking on multiple transcriptome profiles from mutants defective in lignin composition were elaborated to isolate the communities of genes that exhibited expression patterns correlating with the lignin content of the plants. The features included in the so-called lignin-related modules could be followed up to expand our knowledge on new regulatory components on the monolignol biosynthetic pathway.

Besides WGCNA, protein-protein interaction networks (PPINs) emerged as powerful tools to provide holistic interpretations of biological responses based on the protein interplays. Particularly, intra-specific PPINs for plant-pathogen interactions were crucial to expand our understanding about plant immunity, however, completing interactome maps is a task of enormous complexity. Therefore, the computational approach described in the work by Kataria et al. constitutes an inventive example to infer the PPIN for the *Medicago sativa* (alfalfa) and the compatible bacteria *Pseudomonas syringae* patho-system based on predicted interactions. Functional annotation and centrality analysis of proteins included in the host-pathogen PPIN recapitulated a detailed collection of features participating during the infection and highlighted the pivotal role of central defence and signal transduction mechanisms, as well as the prevalent targeting of host chloroplast-located proteins. Furthermore, a series of potential novel *P. syringae* effectors and host targets were proposed. Thus, such studies are necessary to gain insight into the infection and defence strategies and, especially, to address these biological questions in species of agronomical interest, where technical limitations are evident.

Altogether, the works included in this Research Topic highlight the vast potential of system biology and integration of system-wide studies and novel analysis platforms to elucidate and solve key issues in plant biology.

## Author contributions

JA: Writing – original draft, Writing – review & editing. AG-M: Writing – original draft, Writing – review & editing.

